# Pharmacokinetics of praziquantel and its metabolites in grass carp (*Ctenopharyngodon idella*) following the oral administration of a single bolus

**DOI:** 10.17221/109/2023-VETMED

**Published:** 2024-02-27

**Authors:** Radka Dobsikova, Jana Blahova, Petr Marsalek, Veronika Doubkova, Eliska Zuskova, Josef Velisek

**Affiliations:** ^1^Department of Animal Husbandry, Animal Nutrition and Biochemistry, Faculty of Veterinary Hygiene and Ecology, University of Veterinary Sciences Brno, Brno, Czech Republic; ^2^Department of Animal Protection and Welfare and Veterinary Public Health, Faculty of Veterinary Hygiene and Ecology, University of Veterinary Sciences Brno, Brno, Czech Republic; ^3^South Bohemian Research Center of Aquaculture and Biodiversity of Hydrocenoses, Research Institute of Fish Culture and Hydrobiology, Faculty of Fisheries and Protection of Waters, University of South Bohemia in Ceske Budejovice, Vodnany, Czech Republic

**Keywords:** fish, food safety, health, parasites

## Abstract

The study aimed to evaluate and compare the routes and rates of the depletion of the antiparasitic praziquantel (PZQ), a derivative of pyrazinoisoquinoline, following its oral administration in grass carp (*Ctenopharyngodon idella*). We focused on the depletion of PZQ and its major metabolites – *cis*-hydroxy praziquantel (CPZQ) and *trans*-hydroxy praziquantel (TPZQ), in water, the plasma, hepatopancreas, kidney, muscle, and skin, following a single oral administration of PZQ in a concentration of 50 mg/kg. Fish were sampled before the drug administration and then eight times in the course of the 30-day-long experiment. Our results indicate the rapid absorption and elimination of PZQ and its metabolites in all the analysed matrices. The most PZQ-burdened tissue was the hepatopancreas, the gill and the skin. In all the samples, the concentration of the drug and its metabolites consistently declined over time. The residue of the parent compound was detected for the longest time in all the tissues. During the study, a significant (*P* < 0.01) correlation was found within the concentration of PZQ, CPZQ, and TPZQ in the water and all the biological matrices. It was also found that the PZQ residue was not detected below the maximum residue levels (i.e., 20 μg/kg) until 16 days after exposure in the muscle and skin.

In the long term, the most common health problems in farmed fish in the Czech Republic are connected with parasite-induced diseases. The intensity of the infestation and the degree of damage mainly depends on the age and condition of the infected fish including the immune system status, parasite species, high fish density, water quality parameters, etc. (Noga 2010).

The treatment of parasitoses in fish should be justified in terms of the improvement of the health status and fish welfare. The therapeutic use of antiparasitic drugs in fish farms must be safe and pose minimal risk to the fish being treated, to the environment and to those handling the drugs (RUMA guidelines 2010).

Praziquantel (PZQ), a synthetic pyrazinoisoquinoline drug, is effective against a broad spectrum of cestodes (tapeworms) and trematodes (flukes) and is a mainstay of anti-platyhelminth parasite therapy in both human and veterinary medicine (Bader et al. 2019). In the target organism, PZQ disrupts the worm tegument which is characterised by vacuolisation and blebbing (Staudt et al. 1992). Its mechanism of action is connected to the ability to alter the membrane permeability and Ca^2+^ homeostasis through its linkage to voltage-gated Ca^2+^ channels (Meister et al. 2016).

In fish, PZQ can be administered through various routes, i.e., oral, topical and parental (Bader et al. 2019). In intensive aquaculture, the most convenient PZQ administration method is the oral route via the feed as the in-feed administration solution is currently possible to treat a large population of captive fish without stress (Baralla et al. 2020). The recommended concentrations of PZQ administered orally range from 50 and 200 mg/kg and 7 mg/kg to 75 mg/kg for a single dose and a repeated multiple dose, respectively. When used in a bath, PZQ therapeutic doses range from 0.25 mg/l to 50 mg/l according to the bath duration and parasite species. Unfortunately, there is a limited number of studies related to the parental (i.e., injectable) administration of PZQ in fish (Bader et al. 2019).

Many studies have investigated the adverse effects, safety and efficacy of the use of antiparasitics in fish (Kolarova et al. 2022; Velisek et al. 2022; Zuskova et al. 2022), some studies have described the pharmacokinetics of the drugs tested (Soukupova-Markova et al. 2015; Kogiannou and Rigos 2021). However, there is a relative lack of literature resources on the pharmacokinetics of the metabolic products of the parent compounds, especially in regards PZQ.

The pharmacokinetics of PZQ in fish depend on the mode of administration, the drug dose, fish size, environmental conditions, etc. (Hirazawa et al. 2013; Xie et al. 2015).

Depletion studies have been provided for the evaluation of the degradation rate of PZQ in fish, for example, with rainbow trout (*Oncorhynchus mykiss*) following the oral administration of a single dose of 50 mg/kg body weight (Soukupova-Markova et al. 2015), gilthead sea bream (*Sparus aurata*) after 3-days receiving 150 mg/kg (Kogiannou and Rigos 2021), cultured rockfish (*Sebastes schlegelii*) after the oral administration of 200 and 400 mg/kg body weight (Kim et al. 2003), or rice field eels (*Monopterus albus*) fed 10 mg/kg body weight (Xu et al. 2016).

So far, PZQ was not registered for use in aquaculture in Europe, thus its use was only possible in the “off-label” cascade manner regulated by Council Directive 90/676/EEC, Directive 2001/82/EC and Commission Regulation No. 37/2010. Within the drug “off-label” use, a standard withdrawal period of 500 degree days is strictly determined. Nowadays, the maximum residual limit (MRL) of PZQ has been determined for fin fish in the EU legislation. The Commission Implementing Regulation (EU) 2023/981 states that the European Medicines Agency (EMA) Committee for Veterinary Medicinal Products has adopted an opinion recommending the inclusion of praziquantel (the sum of the PZQ isomers) in fin fish into the list of allowed substances within the Annex to Commission Regulation (EU) No. 37/2010. The MRL of PZQ and its isomers in the muscle and skin in natural proportions was set at 20 μg/kg (EMA 2022).

The goal of the study was to investigate the pharmacokinetics including the bioavailability and tissue residues of praziquantel (PZQ) and its major metabolites *cis*-hydroxy praziquantel (CPZQ) and *trans*-hydroxy praziquantel (TPZQ) in grass carp (*Ctenopharyngodon idella*) administered *per* *os* of a single dose of PZQ in a concentration of 50 mg/kg of body weight. The reason for our research was to obtain a wider range of scientific data to evaluate the recently established MRL value. In the course of the study, the PZQ concentration was analysed in the water, blood plasma and selected tissues using liquid chromatography with tandem mass spectrometry. Because limited information is available on pharmacokinetic and depletion studies of PZQ and its major metabolites in particular, our toxicological study will, therefore, fill important knowledge gaps in this area.

## MATERIAL AND METHODS

### Experimental fish

A total of 63 grass carp (*Ctenopharyngodon idella*) individuals aged two years (20.86 cm and 90.54 g of the average total body length and body weight, respectively) were used in the experiment. The fish were obtained from Lnare Fishery, Ltd. (Czech Republic). The test substance was praziquantel (PZQ, 2-cyclohexylcarb-oxo-1,2,3,6,7,11b-hexahydro-4*H*-pyrazino[2,1,-a]isoquinoline) in a powder form (Praziquantel powder, Ecological Laboratories Inc., USA) imported with special permission from the State Veterinary Institute of the Czech Republic.

The experimental fish were randomly divided and kept in three 200-litre aquaria. The selected physical-chemical parameters of the water were monitored every day in all the aquaria. They were as follows: temperature 20.16 ± 0.59 °C, dissolved oxygen > 95%, pH 7.2 ± 0.1, acid neutralisation capacity (ANC_4.5_) 1.10 mmol/l, chemical oxygen demand (COD_Mn_) 7.2 mg/l and a sum of Ca^2+^ and Mg^2+^ 1.03 mg/l.

In addition, the contents of the significant chemical parameters, such as the total ammonia, nitrites, and nitrates, were also continuously monitored. The concentrations during the whole experiment, i.e., during the first four days without a change of water, as well as during the following period with regular water changes, were in the range of 0.01–0.03 mg/l, 0.18–0.26 mg/l, and 45.4–50.9 mg/l, respectively. Before the experiment, the fish were acclimatised to the test conditions for 10 days.

During the test, the fish were fed with the commercial diet Premium Select (Alltech Coppens, The Netherlands) at 4% of the body weight per day. The experiment was carried out at the Research Institute of Fish Culture and Hydrobiology (Vodnany, Czech Republic).

### Treatment and sampling

The first seven fish (20.86 ± 2.60 cm and 90.54 ± 32.30 g total body length and body weight, respectively) were used as the control on day 0 of the experiment (sampling No. 1, before the PZQ administration). Subsequently on day 0, all the 56 remaining experimental fish were administered *per os* PZQ at a single dose of 50 mg/kg live weight using a stomach tube.

Seven fish were then sampled at the consequent sampling times to monitor the drug degradation in the organism.

In the course of the experiment, seven grass carp individuals were sampled on each sampling day as follows: on day 1 (sampling No. 2, 24 h after the PZQ administration), on day (sampling No. 3, 48 h after the PZQ administration), on day 3 (sampling No. 4, 72 h after the PZQ administration), on day 4 (sample No. 5, 96 h after the PZQ administration), on day 10 (sample No. 6, 240 h after the PZQ administration), on day 16 (sample No. 7, 384 h after the PZQ administration), on day 23 (sample No. 8, 552 h after the PZQ administration) and on day 30 (sample No. 9, 720 h after the PZQ administration).

In order to observe the time course of the excretion of PZQ and its metabolites, CPZQ and TPZQ, into the aquatic environment, the experimental fish were kept in a static system, i.e., in aquaria with no change of water, for the first 4 days of the experiment (sampling No. 1–5). Then (sampling No. 6–9), the aquaria water was changed semistatically (once per week) till the end of the experiment.

In the course of each sampling day, blood samples (2 ml, stabilised with 40 IU sodium heparin per 1 ml of blood) were withdrawn from the fish through a cardiac puncture. Thereafter, the fish were euthanised *lege artis* by bleeding after having been stunned by a blow to the head. After killing the fish, tissue samples, i.e., the muscle (fillet without skin), skin (skin from one fillet without scales), gills, hepatopancreas, and kidney, as well as a 1.5 l water sample were obtained for further analysis. The residue of the active substance tested and its metabolites were subsequently analysed by liquid chromatography with tandem mass spectrometry in the laboratory of the Department of Animal Protection and Welfare and Veterinary Public Health (University of Veterinary Sciences Brno, Czech Republic).

### Analytical method of the biological samples

The determination of PZQ and its metabolites (i.e., CPZQ and TPZQ) was performed using high-performance liquid chromatography coupled to triple quadrupole tandem mass spectrometry (LC-MS/MS). The types of samples were analysed as follows: water, plasma, hepatopancreas, kidney, muscle, gill, and skin.

The water samples (1–50 ml depending on analyte concentration) were spiked with isotopically labelled internal standard and further purified by solid phase extraction (SPE). The SPE procedure was performed using SPEC C18 AR cartridges (3 ml, 30 mg; Agilent, USA).

The water sample was passed through a preconditioned cartridge (1 ml methanol and 1 ml water). The cartridge was then washed with water (1 ml), allowed to dry for 10 min and then eluted with acetonitrile (1 ml). The eluted solution was evaporated until dryness under a gentle nitrogen stream, dissolved in deionised water and used for the LC-MS/MS analysis. The blood plasma samples (0.5 ml) were spiked with an isotopically labelled internal standard (5 μl) and purified by solid-phase extraction (same SPE procedure as for the water samples). The eluted solution was evaporated until dryness under a gentle nitrogen stream, dissolved in deionised water and used for the LC-MS/MS analysis. The tissue samples (0.5 g) were spiked with an isotopically labelled internal standard (50 μl) and extracted with methanol (3 ml; 30 s; 800 × *g*) using an immersion blender. The liquid portion was transferred to a glass tube, 3 ml of methanol was added to the rest of the tissue in the homogenisation tube, and the homogenisation was repeated. The pooled homogenates were centrifuged (800 × *g*; 15 min; 20 °C). The supernatant was evaporated until dryness under a gentle stream of nitrogen, dissolved in methanol (20%; v/v) and used for purification by solid-phase extraction (same SPE procedure as for the water samples). The eluate was evaporated until dryness under a gentle stream of nitrogen, dissolved in deionised water and used for the LC-MS/MS analysis.

A Thermo Scientific UHPLC Accela 1250 system was connected to a Thermo Scientific TSQ Quantum Access MAX Triple Quadrupole Instrument (Thermo Scientific, Waltham, MA, USA) equipped with heated electrospray ionisation probe. A Thermo Scientific Hypersil C_18_ (2.1 mm × 50 mm, 1.9 μm) column was used at a constant flow rate of 250 μl/min. The mobile phase consisted of a 0.1% water solution of formic acid (solvent A) and acetonitrile (solvent B). The gradient used was: 0–2.0 min linear gradient from 20% to 90% B; 2.0–7.5 min held at 90% B; 7.5–8.0 min from 90% to 20% B and 8.0–8.5 min held at 20% B for the column to re-equilibrate before the next injection. The full loop injection volume of the sample was set at 2 μl. The heated electrospray ionisation was operated in the positive mode under the following conditions: capillary temperature 350 °C; vaporiser temperature 350 °C; sheath gas pressure 35 psi; auxiliary (drying) gas 10 au; and spray voltage 3 300 V.

The standards were purchased from Toronto Research Chemicals (Canada). All the solvents were purchased from Chromservis, Ltd. (Czech Republic) and were of LC/MS purity (≥ 99.9%). The limit of detection (LOD) determined as a 3 : 1 signal versus noise value were for the water samples as follows: PZQ 0.19 μg/l, CPZQ 0.029 μg/l, and TPZQ 0.26 μg/l. The LODs for the plasma samples were as follows: PZQ 1.9 μg/l, CPZQ 1.2 μg/l, and TPZQ 9.7 μg/l. The LODs for the tissue samples were as follows: PZQ 0.46–0.63 μg/kg, CPZQ 0.77– 0.99 μg/kg, and TPZQ 10–12 μg/kg.

### Ethics

All the procedures complied with the relevant legislative regulations of the Czech Republic (No. 166/1996 and No. 246/1992). The testing was approved by the Ministry of Education, Youth, and Sports of the Czech Republic (Permission No. 3126/2021-3-MSMT). The study did not involve endangered or protected species.

### Statistical analysis

The statistical analysis was performed using Unistat for Excel v6.5 software. The normality and homogeneity of variance were verified by using the Shapiro-Wilk and the Levene tests, respectively. Because the data met the condition of normal distribution, a one-way analysis of variance (ANOVA) followed by Tukey’s post hoc HSD test was used to detect the significant differences between the experimental groups. The relationship between the content of the PRQ and its metabolites in the different tissues was assessed using Spearman’s Rank correlation coefficient.

All the results are presented as the mean ± standard deviation. The significance level for all the statistical analyses was *P* < 0.05. For data below the LOD, half of this value was used for the statistical analysis.

## RESULTS

During the 30-day-long experiment, a total of 9 sets of samples of water, blood plasma and selected tissues, i.e., hepatopancreas, kidney, muscle, gill, and skin, were taken for the evaluation of the PZQ pharmacokinetics including the distribution and elimination rates of the parent drug (i.e., PZQ) and its metabolites (i.e., CPZQ, TPZQ).

Table 1 provides data on the total length and weight of the experimental two-year-old grass carp individuals and the degree days values monitored within the experiment. Degree days are calculated by adding the mean daily water temperature in °C for the total number of days measured. Detailed information on the concentrations of the analysed substances measured in the samples on each sampling day included in the statistical evaluation is given in Tables 2 [Table T3]to 4. In the cases when the concentrations of analytes were found below the LOD, half of this particular LOD was used for the statistical analysis.

**Table 1:** Total length and weight of the experimental grass carp and the degree days values within the experiment

Sampling	Experiment day	Hours after administration	Total length (cm)	Total weight (g)	Degree days (°day)
No. 1	0	0	20.86 ± 2.60	90.54 ± 32.30	0
No. 2	1	24	22.20 ± 0.78	113.43 ± 15.13	18.9
No. 3	2	48	22.00 ± 1.15	100.10 ± 14.96	38.0
No. 4	3	72	23.06 ± 1.98	121.16 ± 26.51	57.4
No. 5*	4	96	21.33 ± 1.87	98.46 ± 20.31	77.3
No. 6*	10	240	21.54 ± 0.88	99.30 ± 14.21	198.8
No. 7*	16	384	21.67 ± 1.61	93.07 ± 18.88	320.5
No. 8*	23	552	19.66 ± 1.30	73.79 ± 13.51	462.5
No. 9*	30	720	20.43 ± 0.74	77.23 ± 9.94	605.6

Values are reported as the mean ± standard deviation

*Sampling No. 5 to 9: after sampling, the water in the aquaria was changed. Degree days are calculated by adding the mean daily water temperature in °C for the total number of days measured

**Table 2:** Results of praziquantel (PZQ) in the tested matrices in the course of the experiment

Day	Water (μg/l)	Plasma (μg/l)	Hepatopancreas (μg/kg)	Kidney (μg/kg)	Muscle (μg/kg)	Gill (μg/kg)	Skin (μg/kg)
0	< 0.19*^c^	< 1.9*^d^	< 0.53*^c^	< 0.63*^b^	< 0.46*^c^	< 0.75*^c^	< 0.55*^c^
1	790.3 ± 36.2 (772.0)^a^	1 368.6** ± **208.1 (1 380.0)^a^	4 318.6** ± **2 455.0 (3 250.0)^a^	1 947.1** ± **636.6 (1 720.0)^a^	709.4** ± **273.2 (716.0)^a^	2 327.1** ± **647.2 (2 400.0)^a^	2 332.9** ± **377.1 (2 220.0)^a^
2	723.3 ± 68.9 (739.0)^ab^	1281.1** ± **275.6 (1 280.0)^ab^	2 742.9** ± **1 614.6 (2 130.0)^ab^	1 535.7** ± **124.2 (1 480.0)^a^	607.3** ± **118.3 (663.0)^ab^	2 114.3** ± **654.3 (2 090.0)^a^	1 784.3** ± **221.5 (1 890.0)^b^
3	689.7 ± 72.9 (668.0)^ab^	1 086.0** ± **184.2 (1 000.0)^b^	1 947.1** ± **335.9 (1 850.0)^b^	1 467.1** ± **438.2 (1 320.0)^a^	645.0** ± **231.7 (556.0)^ab^	2 222.9** ± **496.6 (2 370.0)^a^	1 571.4** ± **192.0 (1 480.0)^b^
4	638.0 ± 32.2 (624.0)^b^	789.9** ± **168.8 (734.0)^c^	2 162.9** ± **331.1 (2 240.0)^b^	1 482.9** ± **580.6 (1 470.0)^a^	433.9** ± **110.6 (413.0)^b^	1 391.9** ± **344.9 (1 560.0)^b^	1 544.3** ± **357.3 (1 500.0)^b^
10	18.0 ± 2.6 (17.7)^c^	29.3** ± **4.8 (27.7)^d^	50.9** ± **6.5 (47.9)^c^	32.1** ± **7.9 (30.7)^b^	20.6** ± **6.5 (17.5)^c^	46.3** ± **29.1 (30.5)^c^	28.8** ± **3.4 (27.6)^c^
16	4.3 ± 0.8 (3.9)^c^	5.6** ± **1.5 (5.7)^c^	14.2** ± **4.1 (13.3)^c^	< 0.63*^b^	3.7** ± **1.0 (3.4)^c^	< 0.75*^c^	< 0.55*^c^
23	0.5 ± 0.1 (0.4)^c^	< 1.9*^d^	1.9** ± **1.3 (2.0)^c^	< 0.63*^b^	< 0.46*^c^	< 0.75*^c^	< 0.55*^c^
30	0.6 ± 0.2 (0.7)^c^	< 1.9*^d^	1.5** ± **0.5 (1.3)^c†^	< 0.63*^b^	< 0.46*^c^	< 0.75*^c^	< 0.55*^c^

^a–d^Statistically significant differences in the concentrations between the sampling days are indicated by different alphabetic superscript letters (*P*** < **0.05); Data are presented as the mean** ± **standard deviation; the median value is given in the brackets; *Results were below the limit of detection, half of this particular limit of detection was used for the statistical analysis; ^†^Descriptive characteristics were calculated only from the positive samples

**Table 3 T3:** Results of *cis*-hydroxy praziquantel (CPZQ) in the tested matrices in the course of the experiment

Day	Water (μg/l)	Plasma (μg/l)	Hepatopancreas (μg/kg)	Kidney (μg/kg)	Muscle (μg/kg)	Gill (μg/kg)	Skin (μg/kg)
0	< 0.029*^g^	< 1.2*^b^	< 0.82*^c^	< 0.99*^b^	< 0.77*^b^	< 1.09*^b^	< 0.83*^d^
1	3.3 ± 0.3 (3.4)^de^	55.2 ± 25.3 (57.8)^a^	117.9 ± 49.2 (119.0)^a^	51.4 ± 17.5 (53.6)^a^	24.3 ± 11.2 (18.1)^a^	139.6 ± 63.5 (154.0)^a^	23.5 ± 7.6 (26.7)^ab^
2	4.2 ± 0.7 (4.1)^cd^	41.1 ± 8.2 (42.2)^a^	72.8 ± 26.4 (68.8)^b^	41.2 ± 7.2 (38.5)^a^	19.9 ± 5.8 (20.7)^a^	133.8 ± 45.3 (142.0)^a^	17.9 ± 4.8 (17.9)^bc^
3	7.4 ± 0.8 (7.8)^b^	45.2 ± 8.7 (43.5)^a^	73.8 ± 16.7 (77.8)^b^	47.3 ± 10.4 (45.5)^a^	18.2 ± 4.9 (18.7)^a^	168.9 ± 42.9 (167.0)^a^	15.6 ± 2.1 (16.4)^c^
4	10.0 ± 0.9 (10.4)^a^	40.1 ± 6.3 (43.1)^a^	88.8 ± 20.1 (95.7)^ab^	27.7 ± 4.6 (26.3)^a^	19.6 ± 7.7 (17.2)^a^	142.3 ± 33.8 (136.0)^a^	27.4 ± 5.6 (24.3)^a^
10	52 ± 1.0 (5.2)^c^	< 1.2*^b^	8.0 ± 1.8 (7.6)^c^	< 0.99*^b^	1.9 ± 0.1 (1.9)^b†^	< 1.09*^b^	< 0.83*^d^
16	1.8 ± 0.2 (1.7)^f^	< 1.2*^b^	5.9 ± 1.0 (6.1)^c^	< 0.99*^b^	< 0.77*^b^	< 1.09*^b^	< 0.83*^d^
23	1.1 ± 0.1 (1.1)^fg^	< 1.2*^b^	2.0 ± 0.3 (1.8)^c†^	< 0.99*^b^	< 0.77*^b^	< 1.09*^b^	< 0.83*^d^
30	0.8 ± 0.3 (0.6)^fg^	< 1.2*^b^	< 0.82*^c^	< 0.99*^b^	< 0.77*^b^	< 1.09*^b^	< 0.83*^d^

^a–g^Statistically significant differences in the concentrations between the sampling days are indicated by different alphabetic superscript letters (*P*** < **0.05); Data are presented as the mean** ± **standard deviation; the median value is given in the brackets; *Results were below the limit of detection, half of this particular limit of detection was used for the statistical analysis; ^†^Descriptive characteristics were calculated only from the positive samples

**Table 4:** Results of *trans*-hydroxy praziquantel (TPZQ) in the tested matrices in the course of the experiment

Day	Water (μg/l)	Plasma (μg/l)	Hepatopancreas (μg/kg)	Kidney (μg/kg)	Muscle (μg/kg)	Gill (μg/kg)	Skin (μg/kg)
0	< 0.26*^d^	< 9.7*^c^	< 10.0*^d^	< 12.0*^b^	< 11.0*^b^	< 11.0*^b^	< 11.0*^c^
1	15.2** ± **1.0 (15.2)^c^	601.6** ± **280.2 (681.0)^a^	426.7** ± **213.1 (411.0)^ab^	473.9** ± **168.5 (439.0)^a^	207.4** ± **75.5 (172.0)^a^	1 440.9** ± **700.5 (1 400.0)^a^	215.8** ± **79.4 (246.0)^a^
2	17.1** ± **2.6 (16.5)^c^	368.0** ± **94.1 (368.0)^b^	312.4** ± **67.4 (317.0)^bc^	283.9** ± **62.5 (263.0)^a^	175.7** ± **34.6 (180.0)^a^	1 161.9** ± **534.3 (1 050.0)^a^	129.9** ± **28.9 (138.0)^b^
3	36.6** ± **4.9 (39.0)^b^	455.6** ± **109.7 (473.0)^ab^	284.9** ± **59.1 (314.0)^c^	370.9** ± **86.3 (372.0)^a^	183.1** ± **49.9 (158.0)^a^	1 356.6** ± **378.1 (1 460.0)^a^	129.1** ± **40.0 (126.0)^b^
4	50.8** ± **5.4 (51.9)^a^	402.6** ± **38.3 (406.0)^b^	458.4** ± **55.8 (462.0)^a^	244.1** ± **49.9 (232.0)^a^	165.9** ± **46.9 (176.0)^a^	1 319.6** ± **294.3 (1 390.0)^a^	195.3** ± **28.8 (183.0)^a^
10	15.6** ± **1.6 (16.5)^c^	< 9.7*^c^	48.4** ± **12.8 (49.2)^d^	< 12.0*^b^	< 11.0*^b^	< 11.0*^b^	< 11.0*^c^
16	1.0** ± **0.2 (0.8)^d^	< 9.7*^c^	13.6** ± **1.9 (13.3)^d^	< 12.0*^b^	< 11.0*^b^	< 11.0*^b^	< 11.0*^c^
23	< 0.26*^d^	< 9.7*^c^	< 10.0*^d^	< 12.0*^b^	< 11.0*^b^	< 11.0*^b^	< 11.0*^c^
30	< 0.26*^d^	< 9.7*^c^	< 10.0*^d^	< 12.0*^b^	< 11.0*^b^	< 11.0*^b^	< 11.0*^c^

^a–d^Statistically significant differences in the concentrations between the sampling days are indicated by different alphabetic superscript letters (*P*** < **0.05); Data are presented as the mean** ± **standard deviation; the median value is given in the brackets; *Results were below the limit of detection, half of this particular limit of detection was used for the statistical analysis; ^†^Descriptive characteristics were calculated only from the positive samples

In the period from the PZQ administration to the water exchange (i.e., experiment day 1 to 4), the highest concentrations of the parent substance PZQ as well as its metabolites CPZQ and TPZQ were logically recorded in the tissues when compared to the term after the water exchange. On day 0 of the experiment, the levels of the analytes of interest were found to be below the detection limit for all the analysed matrices. As for PZQ, its highest concentration was detected in the hepatopancreas (4 318.6 μg/kg on day 1) throughout the exposure. The residue of the parent compound was measured at detectable concentrations in this tissue throughout the experiment. Compared with day 1, the concentration of PZQ in the hepatopancreas was almost 3000-fold lower at the end of the experiment. The other tissues with the highest residue of the parent compound were the gills and skin, with the concentrations of PZQ being the highest on the first day of sampling in both tissues (i.e., 2 327.1 and 2 332.9 g/kg, respectively). Regarding the tissues, the lowest PZQ content was observed in the muscle. Similar to the other tissues, the highest concentration of PZQ in the muscle was recorded on day 1 (709.4 μg/kg).

Within the evaluation of the concentrations of the PZQ metabolites, higher levels were recorded for TPZQ when compared to CPZQ. Surprisingly, a different trend was observed in the tissue occurrence of the highest concentrations compared to the parent compound. For TPZQ, the highest levels were observed in the gill (similar concentrations were found on days 1 to 4 of exposure, ranging from 1 161.9 μg/kg to 1 440.9 μg/kg). However, even in the case of TPZQ (as with PZQ), it was found that within the tissue samples, the residue of TPZQ remained detectable in the hepatopancreas for the longest time (up to day 16 of the experiment). The lowest tissue concentrations of TPZQ were detected in the muscle and skin (207.4 and 215.8 μg/kg, respectively).

The lowest concentrations among all the tissues during the experiment were found for CPZQ. Also, for CPZQ, in agreement with TPZQ, the highest concentrations were found in the gills (ranging from 133.8 μg/kg to 168.9 μg/kg with/in day 1 to 4) and the lowest in the muscle and skin (ranging from 18.2 μg/kg to 24.3 μg/kg and 15.6 μg/kg to 27.4 μg/kg, respectively, within day 1 to 4). Up to 10-fold lower concentrations of CPZQ were found in most tissues compared to TPZQ.

After the exchange of water (i.e., after sampling No. 5), the concentrations of PZQ and its metabolites decreased significantly and were rapidly below the limit of detection (LOD) in most of the analysed samples. The residue was detected in the water, hepatopancreas, and plasma for the longest time. On the other hand, PZQ and its metabolites were removed from the kidney, gill and skin the fastest. Particularly in the case of CPZQ a TPZQ, they were found below the limit of detection (LOD) immediately after the water exchange in these issues. In general, the residue of the original compound, PZQ, were detected the longest in all the tissues throughout the experiment.

As far as the water samples are concerned, a similar trend was observed during the experiment, i.e., the highest concentrations were found for PZQ, followed by TPZQ and CPZQ. The residue of all three monitored analytes were detected in the water almost throughout the experiment.

Another goal of the study was to determine if a correlation existed among the obtained values. The correlation analysis using Spearman’s correlation showed a significant (*P* < 0.01) relationship between the concentration of the drug and its metabolites in all the analysed tissues (data not shown). The correlation coefficients ranged between 0.811 6 (for PRQ in the hepatopancreas vs CPZQ in the gill) and 0.982 0 (for CPZQ in the plasma vs TPZQ in the plasma) which represents a strong positive correlation of the observed parameters.

## DISCUSSION

The study was designed to evaluate the pharmacokinetics of PZQ and its metabolites CPZQ and TPZQ from the water, blood plasma and selected tissues (i.e., hepatopancreas, kidney, muscle, gill and skin) of two-year-old grass carp (*Ctenopharyngodon idella*) which were *per os* administered a single dose of 50 mg/kg PZQ. The results show a rapid increase in the concentration of PZQ and its metabolites within the first 24 h after exposure as well as the quite rapid elimination of the substances from the tissues in the course of the experiment. In general, the study revealed that the water exchange significantly decreases the concentrations of the analytes in the analysed biological samples.

In our study, the plasma concentrations of PZQ in the grass carp administered a single dose of PZQ at a concentration of 50 mg/kg were found similar to the results obtained in the study of Kogiannou et al. (2023) who evaluated single oral PZQ exposition doses (75 and 150 mg/kg) in gilthead seabream (*Sparus aurata*) which resulted in a plasma concentration of PZQ ranging approximately between 1.0 and 2.3 mg/l within 24 h to 96 h after exposure. They also published similar conclusions for PZQ in the gill ranging approximately between 0.1 mg/kg to 2.0 mg/kg. Their experiment shows that the highest concentrations of PZQ in the plasma and gills of gilthead seabream were measured within 4 to 6 h after the PZQ exposure, after which there was a constant decrease in the PZQ levels in the plasma and gills, indicating its rapid rate of removal.

In the study of Xie et al. (2015) using grass carp (*Ctenopharyngodon idella*) for a dietary administration of 10 mg/kg PZQ, the highest PZQ concentration was found in the blood plasma (0.91 mg/l) and muscle (0.62 mg/kg), liver (3.87 mg/kg), and kidney (3.39 mg/kg) within 0.5 h to 1.0 h after exposure. Similarly, to our results, the highest and lowest PZQ concentrations were found in the hepatopancreas and muscle, respectively.

Similar data were published in the study of Xu et al. (2016), in which the oral administration of 10 mg/kg of PZQ for three consecutive days to rice field eel (*Monopterus albus*) resulted in the highest PZQ level in the liver (102.4 μg/kg) and plasma (53.2 μg/l) 48 h after the administration. In their study, PZQ was not detected in the plasma, liver, and muscle 72 h after exposure (as well as in the kidney and skin 96 h after exposure). For comparison, in our study, PZQ residue was found within a much longer time frame after administration, i.e., on day 16 in the plasma and muscle or on day 30 in the hepatopancreas. Also, in the study of Soukupova-Markova et al. (2015), a longer elimination time of PZQ in the hepatopancreas (120 h) and muscle (816 h) was also recorded in rainbow trout (*Oncorhynchus mykiss*) following the oral administration of 50 mg/kg PZQ. The study also states that the concentration of PZQ reached its maximum within 24 h after administration in all the tested samples, and dropped sharply over the subsequent 24 hours.

All the above-mentioned studies proved similar trends in the PZQ pharmacokinetics in the tested fish. However, none considered the depletion rates of PZQ metabolites CPZQ and TPZQ as determined in our study. Moreover, there are no relevant data on the pharmacokinetics of PZQ metabolites in the available professional and scientific databases. Therefore, our study can be considered very important in terms of the bioavailability and degradation of the anthelmintics PZQ and its metabolites CPZQ and TPZQ in fish organisms, as it proved to have significantly (*P*** < **0.01) higher concentrations of TPZQ in all the samples tested when compared to CPZQ. On the other hand, our study revealed that TPZQ, after the water exchange, degraded faster than CPZQ in the plasma and hepatopancreas and muscle tissue. In general, the metabolites were eliminated from the tissues earlier than the parent compound, indicating their faster biodegradability in comparison to PZQ.

In the EU legislation, the maximum residual limit (MRL) of PZQ has been recently determined for fin fish at a concentration of 20 μg**/**kg in the muscle and skin in natural proportions which is very important for the determination of the withdrawal period of PZQ-based anthelmintics used in aquaculture. That is why the study also aimed to determine how long it takes to eliminate the administered amount of PZQ from the muscle and skin of grass carp to decrease the drug residue concentration below the determined MRL. It was found that PZQ residues were not determined below the MRL until 16 days after exposure in both tissues (i.e., muscle and skin) of grass carp. Another important finding is that PZQ metabolites were present in muscle and skin in detectable amounts for a shorter period than the parent compound.

The results of our study may help to raise awareness of the pharmacokinetics of the drug and its metabolic products in individual tissues of farmed fish species and can be used to establish the withdrawal period of PZQ-based anthelmintics used in aquaculture.
